# Identification of Volatile Organic Compounds in Extremophilic Bacteria and Their Effective Use in Biocontrol of Postharvest Fungal Phytopathogens

**DOI:** 10.3389/fmicb.2021.773092

**Published:** 2021-11-12

**Authors:** Laura Toral, Miguel Rodríguez, Fernando Martínez-Checa, Alfredo Montaño, Amparo Cortés-Delgado, Agnieszka Smolinska, Inmaculada Llamas, Inmaculada Sampedro

**Affiliations:** ^1^Xtrem Biotech S.L., European Business Innovation Center, Avenida de la Innovación, Granada, Spain; ^2^Department of Microbiology, Faculty of Pharmacy, Campus de Cartuja s/n, Granada, Spain; ^3^Biomedical Research Center (CIBM), Avenida del Conocimiento s/n, Granada, Spain; ^4^Department of Food Biotechnology, Instituto de la Grasa, Sevilla, Spain; ^5^Department of Pharmacology and Toxicology, Maastricht University, Maastricht, Netherlands

**Keywords:** volatile compounds, antifungal activity, biocontrol, fungal phytopathogens, postharvest diseases

## Abstract

Phytopathogenic fungal growth in postharvest fruits and vegetables is responsible for 20–25% of production losses. Volatile organic compounds (VOCs) have been gaining importance in the food industry as a safe and ecofriendly alternative to pesticides for combating these phytopathogenic fungi. In this study, we analysed the ability of some VOCs produced by strains of the genera *Bacillus*, *Peribacillus*, *Pseudomonas*, *Psychrobacillus* and *Staphylococcus* to inhibit the growth of *Alternaria alternata*, *Botrytis cinerea*, *Fusarium oxysporum*, *Fusarium solani*, *Monilinia fructicola*, *Monilinia laxa* and *Sclerotinia sclerotiorum*, *in vitro* and *in vivo*. We analysed bacterial VOCs by using GC/MS and 87 volatile compounds were identified, in particular acetoin, acetic acid, 2,3-butanediol, isopentanol, dimethyl disulphide and isopentyl isobutanoate. *In vitro* growth inhibition assays and *in vivo* experiments using cherry fruits showed that the best producers of VOCs, *Bacillus atrophaeus* L193, *Bacillus velezensis* XT1 and *Psychrobacillus vulpis* Z8, exhibited the highest antifungal activity against *B. cinerea*, *M. fructicola* and *M. laxa*, which highlights the potential of these strains to control postharvest diseases. Transmission electron microscopy micrographs of bacterial VOC-treated fungi clearly showed antifungal activity which led to an intense degeneration of cellular components of mycelium and cell death.

## Introduction

The world population has increased by 1 billion over the last 10years, reaching a total of 7.8 billion currently, which is expected to rise by a further 1 billion by 2030. Regardless of environmental damage and potential risks to human health, the use of chemical fertilisers in agriculture and during postharvest storage has increased and remains at high levels given the growing demand for food worldwide (Goswami and Deka, 2020). The factors responsible for postharvest losses include fungal pathogen infections which are estimated to account for approximately 20–25% of fruit and vegetable postharvest decay in developed countries and contribute significantly to a deterioration in quality and nutrient composition, mycotoxin contamination and a reduction in the market value of fruit (Frankowski et al., 2001; Mari et al., 2016; Gotor-Vila et al., 2017; Tozlu et al., 2018). Many fungal species of the most diverse genera have been reported to be associated with postharvest diseases in fruits and vegetables worldwide. These include *Penicillium expansum*, *Penicillium italicum* and *Penicillium digitatum* (Cheng et al., 2020; Kanashiro et al., 2020; Yu et al., 2020; Zhang et al., 2020a), *Alternaria alternata* (Tozlu et al., 2018; Garganese et al., 2019), *Phytophthora citrophthora* (Díaz et al., 2020) the necrotrophic pathogens *Sclerotinia sclerotiorum* and *Botrytis cinerea* (Fernando et al., 2005; Giorgio et al., 2015; Massawe et al., 2018; Zhao et al., 2020), *Colletotrichum gloeosporioides* (Jin et al., 2020; Shi et al., 2021), various species belonging to the genus *Fusarium* (Medina-Romero et al., 2017; Go et al., 2019; Lee et al., 2019) and the genus *Monilinia*, known to be the most important fungal pathogen to infect stone fruits (Martini and Mari, 2014; Rungjindamai et al., 2014; Obi et al., 2018).

Although postharvest decay has traditionally been controlled through chemical fungicides, their intensive use can cause problems, such as pathogen resistance, pesticide residues, human health hazards and environmental pollution (Mari et al., 2016; Lim et al., 2017). Moreover, due to the toxicological risks involved, chemicals registered for postharvest use are severely limited while consumer awareness of the need for pesticide-free food has been increasing (Gao et al., 2017; Lastochkina et al., 2020). Thus, given their safe, ecofriendly and sustainability properties, biopesticides, which meet the global strategic requirements of organic agriculture, could be a desirable alternative to traditional pesticides (Wu et al., 2019; Zheng et al., 2019).

A wide range of ecological strategies has emerged with the use of anti-phytopathogenic microorganisms and the use of plant-defence hormones or glucosinolates (Poveda, 2020; Poveda et al., 2020). Given the variety of possible fungal inhibition pathways and the wide range of microbial secondary metabolites, postharvest disease management involving biocontrol agents (BCAs) has, in recent years, focused on the use of volatile organic compounds (VOCs; Ryu et al., 2004; Guevara-Avendano et al., 2019). Species of the genera *Streptomyces*, *Pseudomonas*, *Serratia*, *Xanthomonas*, *Alcaligenes*, *Bacillus* and *Agrobacterium* are reported to be the most frequent producers of these bioactive VOCs (Chaves-Lopez et al., 2015; Schmidt et al., 2015; Asari et al., 2016; Mari et al., 2016; Dias et al., 2017; Gotor-Vila et al., 2017; Lim et al., 2017; Khan et al., 2018).

Microbial volatile organic compounds (MVOCs), which originate from different metabolic pathways during fungal and bacterial growth, contain low molecular weight and high vapour pressure molecules that readily diffuse through water and gas-filled pores in soil environments (Chaves-Lopez et al., 2015; Parafati et al., 2017; Toffano et al., 2017). From a control perspective, these characteristics expand the area of influence, improve membrane penetration and consequently enhance the lethality of these microorganisms (Logan et al., 2009). Bacterial VOCs inhibit spore germination and mycelial growth of various phytopathogens, promote plant growth and induce plant resistance (Gotor-Vila et al., 2017; Lazazzara et al., 2017; Syed-Ab-Rahman et al., 2019; Di Francesco et al., 2020; Poveda, 2021). However, the composition and antifungal properties of volatiles produced by microorganisms can vary according to the growing medium, oxygen availability, moisture, temperature and pH, as well as the population involved and functional dynamics (Chaves-Lopez et al., 2015; Schmidt et al., 2015). Moreover, MVOCs produced by BCAs can play different regulatory roles in different species, the extent of whose inhibition depends on specific bacterium-fungus interactions (Schmidt et al., 2015; Zheng et al., 2019).

This study thus aims to evaluate the antifungal activity of VOCs produced by bacteria obtained from extreme environments and belonging to the genera *Peribacillus*, *Pseudomonas*, *Staphylococcus*, *Psychrobacillus* and *Bacillus* against seven postharvest fruit pathogens. (i) An *in vitro* approach, together with scanning and transmission microscopy, was used to evaluate the antifungal effect of antipathogenic bacterial VOCs on colony and mycelial growth; (ii) VOCs were identified using headspace solid-phase microextraction coupled with gas chromatography-mass spectrometry (HS-SPME-GC/MS); (iii) the effect of pure compounds on the target pathogens was tested *in vitro*; and (iv) the impact of antifungal activity of bacterial VOCs and synthetic compounds was assayed *in vivo* on fruits. The impact of the culture medium on VOCs composition and its antifungal activity was also evaluated.

## Materials and Methods

### Bacterial and Fungal Strains, Growth Media and Culture Conditions

The bacterial strains used in this study had been isolated from different sources: *Peribacillus* sp. N3 from river otter (*Lutra lutra*) faeces and strains *Bacillus atrophaeus* L193 (Rodriguez et al., 2018), *Bacillus velezensis* XT1, a patented strain (Béjar et al., 2014), *Pseudomonas segetis* P6 (Rodriguez et al., 2020b), *Psychrobacillus vulpis* Z8 (Rodriguez et al., 2020a) and *Staphylococcus equorum* subsp. *equorum* EN21 (Vega et al., 2019) from saline and hypersaline environments. We examined the phytopathogenic fungi *A. alternata* CECT 20560, *B. cinerea* (isolated from *Vitis vinifera* L. and kindly provided by the Plant Food Research Group, University of Zaragoza, Spain), *Fusarium oxysporum* CECT 2159, *Fusarium solani* [isolated from *Solanum tuberosum* and kindly provided by the Andalusian Agricultural and Fisheries Research and Training Institute (IFAPA), Cordoba, Spain], *Monilinia fructicola*, *Monilinia laxa* (both isolated from *Prunus persica* L. and kindly provided by the Plant Food Research Group, University of Zaragoza, Spain) and *S. sclerotiorum* CECT 2769.

*Bacillus velezensis* strain XT1 was cultured in nutrient broth (NB) medium (No. 3 NutriSelect^®^ Plus, Merck) while the other bacterial strains were cultured in tryptic soy broth (TSB, Panreac^®^ Applichem) at 28°C and 120rpm in a rotary shaker unless otherwise stated. Fungal strains were cultured in potato dextrose agar (PDA, Difco^®^) medium at 21°C. To produce bacterial volatile compounds, strains were cultured in tryptic soy agar (TSA, Panreac^®^ Applichem) medium, Schaeffer’s growth (SG) medium (Kovacs, 1928) and the medium optimal for lipopeptide production (MOLP; Ahimou et al., 2000). The pH of each medium was adjusted to 7.2–7.4 using NaOH 1M or HCl 1N.

### Gas Chromatography Analysis of Bacterial Volatile Compounds

The bacterial strains were cultured in flask containing 200ml of MOLP, SG or TSB for 24h at 28°C and 120rpm, and the volatile compounds produced were analysed using HS-SPME-GC/MS according to the procedure described by Montaño et al. (2021). Uninoculated MOLP, SG and TSB samples were analysed as controls in order to remove natural occurring volatile compounds from each medium. An aliquot of each sample (5ml) was inserted into a 15ml glass vial and 50μl of internal standard (5-nonanol; 2mgL^−1^) was added. The vial was closed and placed in a water bath adjusted to 40°C and stirred at 600rpm using a stirring bar. After 15min of equilibration, the headspace volatile compounds were extracted for 30min using a divinylbenzene/carboxen/polydimethylsiloxane (DVB/CAR/PDMS) fibre (2cm, 50/30μm; Supelco, Bellefonte, PA). The volatiles were then desorbed for 15min at 265°C in a GC injector port interfaced with a mass detector with a scan range of m/z 30–400. Separation was carried out on a VF-WAX MS capillary column (30m×0.25mm×0.25μm thickness film) from Agilent Technologies (Santa Clara, CA, United States). The initial oven temperature was 40°C (5min), followed by 40–195°C at 3°C min^−1^ and then held at 195–240°C at 10°C min^−1^ for 15min. Helium was used as the carrier gas at 1mlmin^−1^ constant flow. Data processing was carried out using MassHunter software (Agilent Technologies). The volatile compounds were initially identified by comparing MS peaks to those in the NIST 17 MS library. The results were then confirmed by comparing the retention indices to literature data reported for equivalent columns and to authentic standards when available. The volatile compounds were quantified by comparison of peak areas to that of internal standard (5-nonanol). All analyses were done in duplicate (i.e., two vials for each bacterial strain).

### *In vitro* Antifungal Activity of Bacterial Volatile Compounds

The effects of bacterial volatile compounds on the mycelial growth of the fungal phytopathogens *A. alternata*, *B. cinerea*, *F. oxysporum*, *F. solani*, *M. fructicola*, *M. laxa* and *S. sclerotiorum* were assessed using the bi-plate Petri dish method (Lim et al., 2017), as well as MOLP, SG and TSA media to assess bacterial growth. Briefly, 5μl from an overnight culture (10^8^CFUml^−1^) of each bacterial strain in NB or TSB was spotted in the centre of one of the bi-plate compartments. In the other compartment containing the PDA medium, a mycelium plug (Ø 5mm) of each fungus from a 15day culture was removed using a sterile cork borer and deposited on the agar bi-plates. The plates were immediately sealed with a double layer of Parafilm to prevent volatile leakage and then incubated at 28°C for 24h followed by incubation at 21°C for 15days. Antifungal activity was measured by the percentage reduction in the mycelial growth with the aid of ImageJ software (Schneider et al., 2012). The experiments were repeated in triplicate using bi-plates with sterile liquid medium instead of bacterial inoculum as control for fungal growth.

### *In vivo* Biocontrol of Fungal Phytopathogens by Bacterial and Synthetic Volatile Compounds

*In vivo* antifungal activity of bacterial volatile compounds, together with the main synthetic compounds identified by GC/MS, was analysed in cherry fruits (*Prunus avium* cv. Picota) according to the protocol described by Gotor-Vila et al. (2017) and Gao et al. (2018), since cherry fruits are the shared host for all fungal pathogens tested. Briefly, cherries were tap-washed and surface-sterilised by spraying with 1% (w/v) sodium hypochlorite solution followed by 70% (v/v) ethanol and sterile distilled water. Each fruit was wounded using a sterile scalpel, and 10μl of each fungal suspension (10^6^ spore ml^−1^) was deposited. The fruits were placed in 2L (13×13×12cm) plastic boxes with 20ml of sterile water-soaked medical gauze (13×13cm). Three uncovered Petri dishes containing MOLP medium for each bacterial strain were placed inside the boxes and incubated at 21°C for 7days. The same protocol was used for testing the synthetic volatile compounds acetic acid, acetoin, 2,3-butenodiol, dimethyl disulphide (DMDS), isopentanol and isopentyl isobutanoate using 10ml glass vials containing 5ml of a solution of each compound (50μm) in sterile distilled water. Disease incidence and symptom severity were then determined. Experiments were carried out in triplicate using five fruits per replicate; negative controls consisted of a box containing Petri dishes with uncultured media.

### Microscopic Analysis of Structural Effects of Volatile Compounds

Fungal morphology following bacterial VOC treatments (bi-plate method) was studied using a transmission electron microscopy (TEM) high-resolution FEI Titan G2 50-300 microscope equipped with a high angle annular dark field detector. For this purpose, mycelium blocks were cut into 1×1mm pieces, fixed with 2.5% glutaraldehyde in phosphate buffer (pH 7.2), dehydrated with an ethanol gradient and embedded in Epon 812 resin. Thin sections (70nm) were cut with a diamond knife (Leica, EM UC7, Germany) and stained with 2% uranyl acetate for 10min, followed by 3% lead citrate for 3min.

Untreated fungal samples were used as negative controls for comparative purposes.

### Statistical Analysis

The Shapiro–Wilk test was used to verify data normality, and the data were statistically analysed with the aid of the ANOVA (*p*≤0.05) and Tukey tests using SPSS software. In order to detect any groupings of volatile compounds based on the composition of the culture media and the strains, as well as to identify the main components of each group, the data were subjected to principal component analysis (PCA) using SIMCA software version 14.1 (Umetrics, Sweden).

## Results

### Characterisation of Bacterial Volatile Compounds Using GC/MS

The VOCs produced by the six bacterial strains, *B. atrophaeus* L193, *B. velezensis* XT1, *Peribacillus* sp. N3, *P. segetis* P6, *P. vulpis* Z8 and *S. equorum* subsp. *equorum* EN21, grown in 24h cultures, were identified and quantified using GC/MS following headspace solid-phase microextraction (HS-SPME). The culture media MOLP, SG and TSA were tested in order to determine the impact of their composition on the production of VOCs and their involvement in antifungal activity. A total of 87 compounds were identified after analysing the uninoculated sterile media and each strain culture in all media (Supplementary Table S1). Butanal, methyl acetate and 2-methyl-2-butenal compounds, which were found exclusively in MOLP and TSB uninoculated sterile media, were not also detected in the cultured media. The remaining 84 compounds were identified as ketones (21.4%), esters (21.4%), alcohols (15.5%), carboxylic acids (11.9%), sulphur compounds (11.9%), aromatic hydrocarbons (10.7%), aldehydes (4.8%), halogenated compounds (1.2%) and terpens (1.2%).

**Figure 1:**
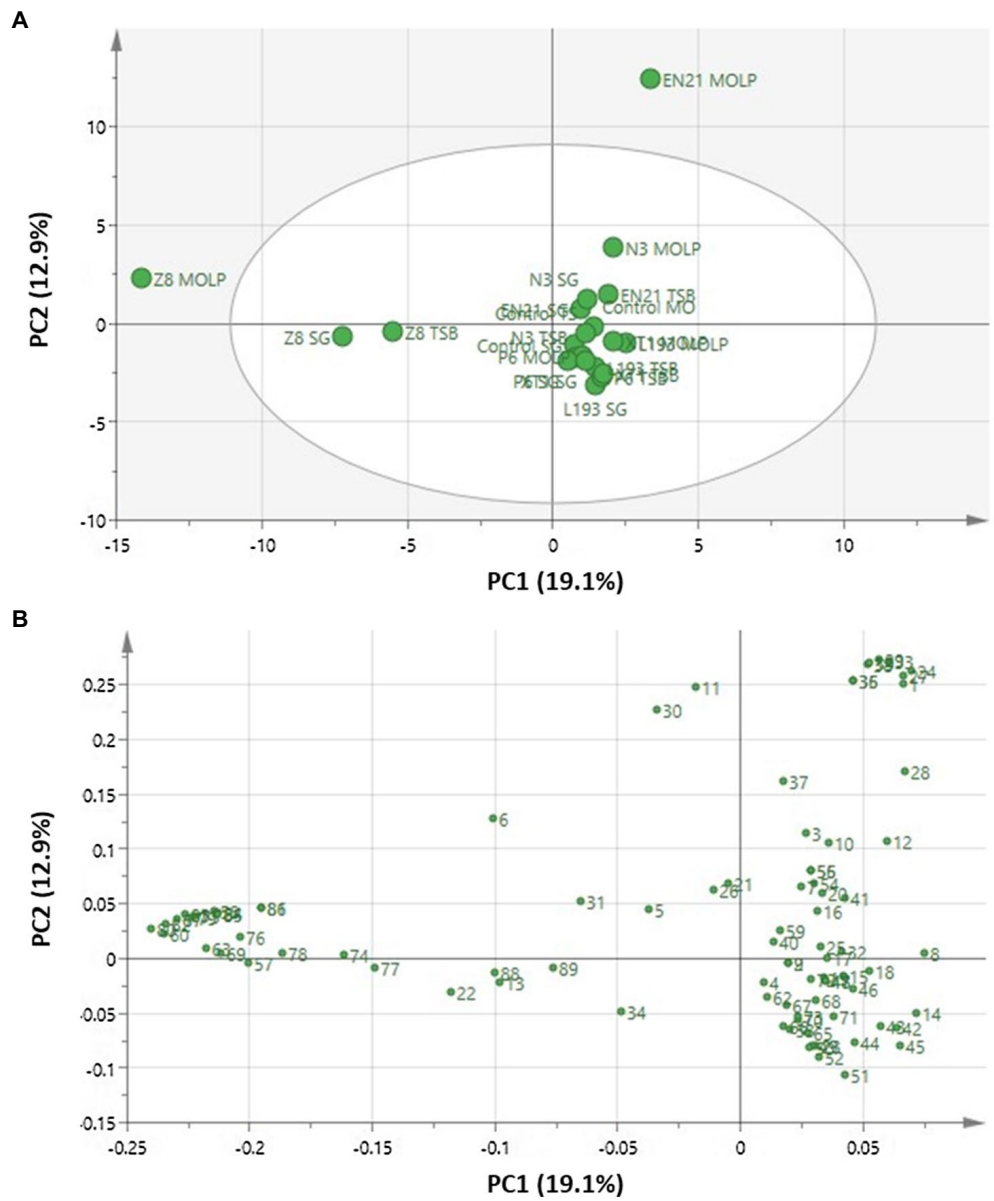
Principal component analysis of volatile compounds obtained using HS-SPME and identified with the aid of GC/MS in *B. atrophaeus* L193 (L193), *B. velezensis* XT1 (XT1), *Peribacillus* sp. N3 (N3), *P. segetis* P6 (P6), *S. equorum* EN21 (EN21) and *P. vulpis* Z8 (Z8) grown in MOLP, SG and TSB media, as well as in uncultured control media. **(A)** Score plot. **(B)** Loading plot. Each volatile compound, which is assigned a reference number, can be seen in Supplementary Table S1.

Principal component analysis was used to detect VOCs clustering and to determine the relationships between the bacterial strains and culture media. The principal components (PCs), which accounted for the largest variations in data points, were extracted in order to better visualise the data structure in a reduced dimension. The overall PCA dataset consisted of a 21×87 matrix, with rows representing 21 bacterial cultures and uncultured media, as well as columns representing volatile compound concentrations. General PCA (Figure 1) revealed two main components which, as indicated by the score plot, accounted for 32% of total variance (Figure 1A). 19.1% of variance was attributable to principal component 1 (PC1), while 12.9% of variance corresponded to PC2. All culture strains clearly formed a highly correlated group, except for strain *P. vulpis* Z8 cultured in MOLP, SG and TSB media and strain *S. equorum* EN21 cultured in MOLP medium, which were segregated from the main group. The PCA loading plot (Figure 1B) shows that this segregation is mainly associated with the volatile compounds butyl isobutanoate (80), isopentyl isobutanoate (82), methanethiol (60), 2-phenylethyl isobutanoate (87), S-methyl thio-3-methylbutanoate (83), butyl propanoate (79) and ethyl 2-methylbutanoate (75) produced by strain *P. vulpis* Z8 for PC1, while PC2 was mainly associated with the volatile compounds propanoic acid (29), butyl acetate (33), hexanoic acid (38), octanoic acid (39), acetic acid (24), butanoic acid (27), 2,6-diethyl-pyrazine (35) and acetone (1) produced by strain *S. equorum* EN21 in the MOLP medium. These volatile compounds were mostly or exclusively released from stains *P. vulpis* Z8 and *S. equorum* EN21 and at very low levels from other strains.

In order to identify the impact of culture media composition on volatile compound production, each bacterial strain was cultured in MOLP, SG and TSB media. Strain *B. atrophaeus* L193 produced 25, 22 and 25 volatile compounds in MOLP, SG and TSB media, respectively, most of which were ketones and aromatic hydrocarbons. In the case of strain *B. velezensis* XT1, the 27, 16 and 27 volatile compounds in MOLP, SG and TSB media, respectively, also mainly corresponded to ketones and aromatic hydrocarbons. With regard to strain *Peribacillus* sp. N3, a total of 37, 20 and 23 compounds were identified in MOLP, SG and TSB media, respectively, most of which were carboxylic acids and ketones. Strain *P. segetis* P6 produced 21, 10 and 21 volatile compounds, mostly ketones, aromatic hydrocarbons and sulphur compounds, in MOLP, SG and TSB media, respectively. In the case of strain *S. equorum* EN21, a total of 28, 18 and 21 volatile compounds, mostly alcohols, carboxylic acids and aromatic hydrocarbons, were detected in MOLP, SG and TSB media, respectively. Finally, with regard to strain *P. vulpis* Z8, a total of 40, 22 and 29 compounds, for the most part esters and sulphur compounds, were identified in MOLP, SG and TSB cultures, respectively.

The main volatile compounds produced by each strain were then selected on the basis of two criteria: their absence from uncultured media and a 2-fold increase in their concentrations in uncultured media. The volatile compounds selected are shown in Table 1. High levels of acetoin and 2,3-butanediol were produced by strains *B. atrophaeus* L193 and *B. velezensis* XT1. Acetoin was synthesised in all media, with particularly high levels of production in MOLP, while 2,3-butanediol was only detected in MOLP cultures. Acetic acid was the principal volatile compound detected when strains *Peribacillus* sp. N3 and *S. equorum* EN21 were cultured in MOLP and SG media. High levels of isopentanol were also produced by *S. equorum* EN21 culture in all media, especially TSB. DMDS was the main volatile compound produced by strain *P. segetis* P6 when cultured in SG medium, as well as by strain *P. vulpis* Z8 when cultured in all media. High concentrations of isopentyl isobutanoate were only detected when strain *P. vulpis* Z8 was cultured in all media.

**Table 1:** Principal volatile compounds for each strain after culturing in MOLP, SG and TSB media.

Sample	Culture medium	Concentration of principal volatile compounds (μgL^−1^)					
		Acetoin	Acetic acid	2,3-butanediol	Isopentanol	Dimethyl disulphide	Isopentyl isobutanoate
Uninoculated media	MOLP	–	3.8 (3.0)	–	–	–	–
	SG	–	–	–	–	–	–
	TSA	–	–	–	–	–	–
*B. atrophaeus* L193	MOLP	174.0 (10.4)	36.9 (7.1)	268.8 (23.0)	–	–	–
	SG	60.5 (15.6)	10.0 (6.5)	–	–	–	–
	TSA	44.5 (0.3)	3.6 (1.1)	–	–	–	–
*B. velezensis* XT1	MOLP	111.0 (8.5)	21.9 (0.4)	261.7 (49.3)	–	–	–
	SG	64.0 (9.6)	6.1 (4.1)	–	–	–	–
	TSA	60.0 (0.5)	2.1 (0.0)	–	–	–	–
*Peribacillus* sp. N3	MOLP	–	32.9 (4.5)	–	7.7 (0.2)	–	–
	SG	–	30.8 (0.4)	–	–	–	–
	TSA	–	–	–	–	5.6 (1.8)	–
*P. segetis* P6	MOLP	–	–	–	–	3.6 (0.3)	–
	SG	–	–	–	–	46.1 (2.9)	–
	TSA	–	–	–	3.1 (0.1)	–	–
*P. vulpis* Z8	MOLP	–	–	–	6.1 (1.1)	88.4 (2.7)	82.5 (1.3)
	SG	–	–	–	–	139.7 (1.5)	27.4 (1.9)
	TSA	–	–	–	–	140.1 (4.3)	52.0 (1.1)
*S. equorum* subsp. *equorum* EN21	MOLP	–	180.2 (17.1)	–	34.3 (2.1)	–	–
	SG	–	25.6 (1.9)	–	73.4 (3.5)	–	–
	TSA	–	4.1 (2.0)	–	110.6 (27.7)	–	–

*Concentrations of each volatile compound are expressed as the mean of two determinations, with standard deviation (SD) indicated in parenthesis – concentration not detected or insignificant*.

Principal component analysis based on intraspecific differences in medium composition showed a distribution very similar to that previously obtained even though a higher level of correlation was observed for PC1 and PC2 in each medium (Supplementary Figure S1).

### *In vitro* Antifungal Activity of Bacterial Volatile Compounds

The effects of VOCs on the mycelial growth of fungal phytopathogens *A. alternata*, *B. cinerea*, *F. oxysporum*, *F. solani*, *M. fructicola*, *M. laxa* and *S. sclerotiorum* were assessed using the bi-plate Petri dish method, in the three-culture media assayed previously for VOC characterisation.

**Figure 2:**
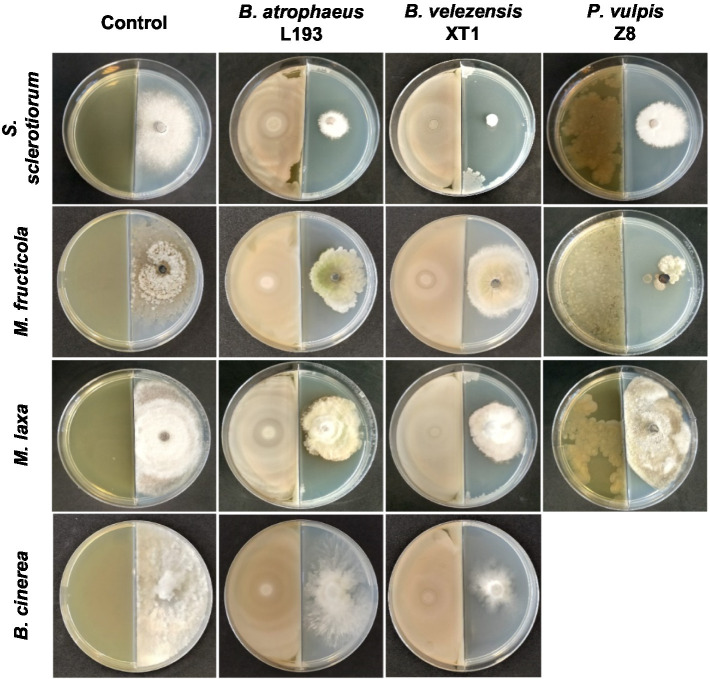
*In vitro* fungal growth inhibition by bacterial volatile compounds produced by *B. atrophaeus* L193, *B. velezensis* XT1 and *P. vulpis* Z8 when cultured in MOLP medium using the bi-plate method.

The highest antifungal activity was observed when strains were cultivated in the MOLP medium. Thus, volatile compounds produced by strains *B. atrophaeus* L193, *B. velezensis* XT1 and *P. vulpis* Z8 synthesised in the MOLP medium greatly inhibited the growth of *S. sclerotiorum* (82, 96 and 56%, respectively), *M. fructicola* (42, 37 and 83%) and *M. laxa* (51, 54 and 15%). With regard to *B. cinerea* plates, *B. atrophaeus* L193 and *B. velezensis* XT1 produced reductions of 27 and 46% in fungal growth, respectively, while *P. vulpis* Z8 shows no effect on the mycelial growth of *B. cinerea* (data not shown). Fungal growth inhibition of some bacterial strains cultured in the MOLP medium is shown in Figure 2. By contrast, no inhibition of mycelial growth was detected against *A. alternata*, *F. oxysporum* and *F. solani* following exposure to VOCs produced by the bacterial strains.

### *In vivo* Biocontrol of Fungal Phytopathogens by Bacterial Volatile Compounds

**Figure 3:**
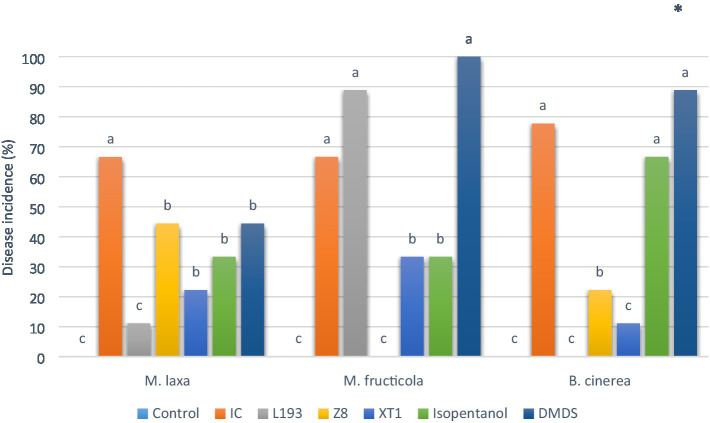
*In vivo* antagonistic activity of volatile organic compounds (VOCs) from *B. atrophaeus* L193, *B. velezensis* XT1 and *P. vulpis* Z8, as well as the synthetic compounds isopentanol and dimethyl disulphide. The figure shows the disease incidence of cherry fruits artificially inoculated with *M. laxa*, *M. fructicola* and *B. cinerea*. Control: control treatment without bacterial VOCs or volatile synthetic compounds. IC: pathogen infection control without VOC treatment. Differences between treatments were tested for statistical significance using Chi-squared test: ^*^(*p*≤0.05), ^**^(*p*≤0.01), ^***^(*p*≤0.001) and ns (not significant).

To examine *in vivo* antifungal activity, VOCs produced by strains *B. atrophaeus* L193, *B. velezensis* XT1 and *P. vulpis* Z8 cultured in the MOLP medium were assayed in cherry fruits, together with some of the principal synthetic volatile compounds characterised by GC/MS. With regard to the percentage of disease incidence, the pathogen *M. laxa* was susceptible to VOCs produced by the strains *B. atrophaeus* L193 and *B. velezensis* XT1 (Figure 3). In both cases, the VOCs reduced disease incidence by over 50% as compared to the control. For its part, no significant antifungal activity against *M. laxa* was produced by *P. vulpis* Z8 VOCs. Similar results were obtained with regard to the synthetic volatile compounds isopentanol and DMDS. A very different scenario was observed for antifungal activity against the pathogen *M. fructicola*, where VOCs produced by *B. atrophaeus* L193 had no effect, while *P. vulpis* Z8 completely inhibited this pathogen’s growth. The VOCs produced by strain *B. velezensis* XT1, and the synthetic compound isopentanol, reduced disease incidence in cherries by 50% as compared to the infection control, while the pathogen *B. cinerea* was completely inhibited by *B. atrophaeus* L193 VOCs. Similarly, strains *P. vulpis* Z8 and *B. velezensis* XT1 reduced the incidence of *B. cinerea* by 48 and 62%, respectively. By contrast, isopentanol and DMDS showed no antifungal activity against *B. cinerea*. The quantity of spores from all pathogenic fungi on the surface of fruits was small and limited to the wound observed when *B. atrophaeus* L193, *B. velezensis* XT1 and *P. vulpis* Z8 VOCs were tested (Supplementary Figure S2). On the other hand, while isopentanol and DMDS had no effect on disease sporulation of *M. fructicola* or *B. cinerea*, they did have a slight impact on *M. laxa*. The synthetic compounds acetoin, acetic acid, 2,3-butanediol and isopentyl isobutanoate did not reduce the disease incidence or fungal sporulation of any target pathogens.

### Microscopic Analysis of Structural Effects of Volatile Compounds

**Figure 4:**
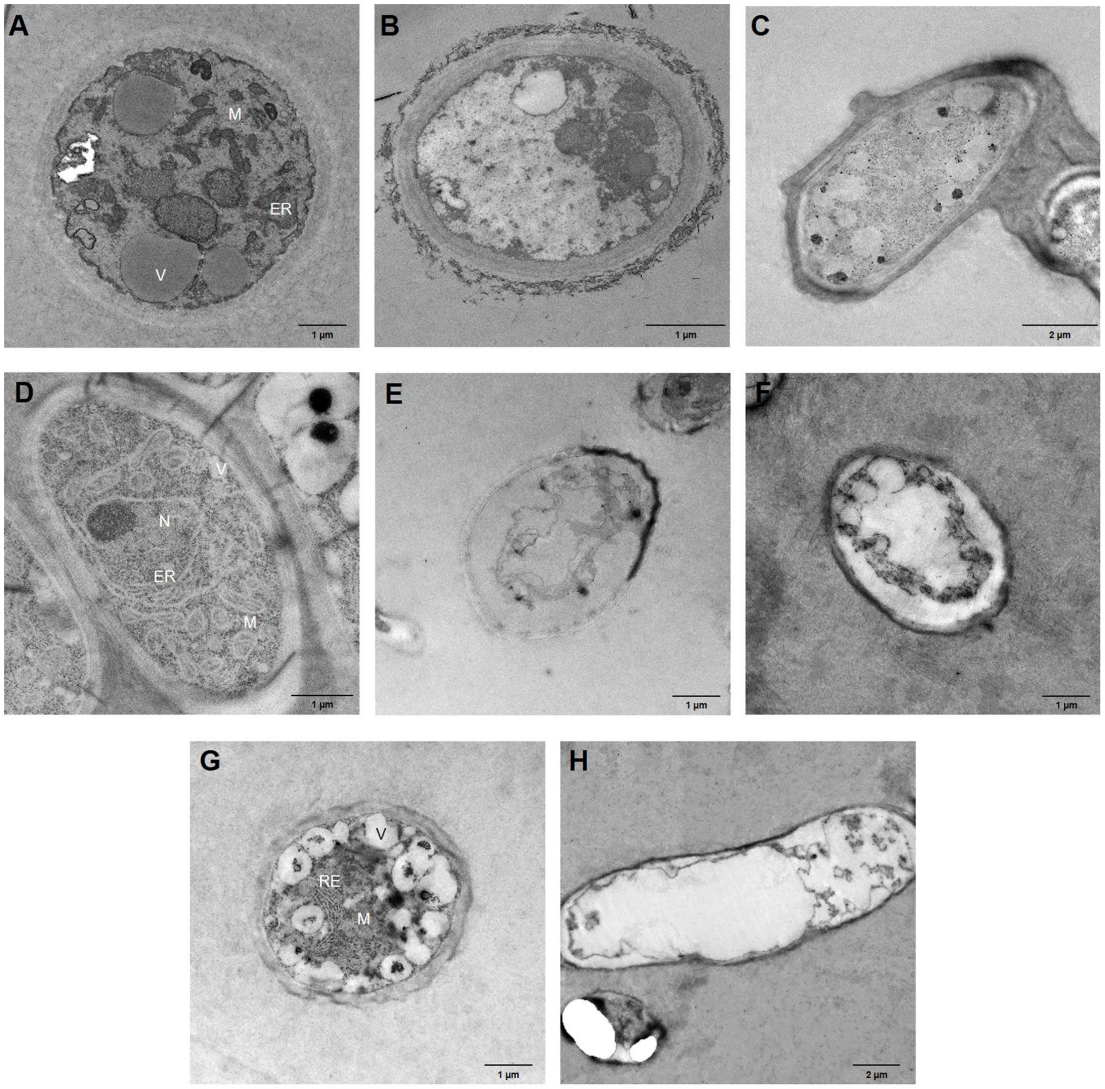
Transmission electron micrographs of fungal hyphae after 7days of bacterial VOC treatment. **(A)** Untreated *B. cinerea*. **(B)** *B. cinerea* – *B. velezensis* XT1 strain. **(C)** *B. cinerea* – *B. atrophaeus* L193 strain. **(D)** Untreated *M. laxa*. **(E)** *M. laxa* – *B. velezensis* XT1 strain. **(F)** *M. laxa* – *B. atrophaeus* L193 strain. **(G)** Untreated *M. fructicola*. **(H)** *M. fructicola* – *P. vulpis* Z8 strain. ER, endoplasmic reticulum; M, mitochondria; N, nucleus; and V, vacuole.

To identify structural disorders caused by bacterial VOC treatment of the phytopathogenic fungi, hyphae were analysed after a 7day incubation period using TEM. Micrographs of control fungi show well-organised cell walls, as well as cellular membranes, while organelles, such as endoplasmic reticula, abundant mitochondria, nuclei, and vacuoles, can be clearly observed in most of these micrographs (Figures 4A,D,G). By contrast, while maintaining the cell wall structure, *B. cinerea* hyphae treated with *B. velezensis* XT1 (Figure 4B) and *B. atrophaeus* L193 volatiles (Figure 4C) showed severe cytoplasmic cavitation and vacuolation and no organelles were identified. When *M. laxa* was exposed to *B. velezensis* XT1 (Figure 4E) and *B. atrophaeus* L193 (Figure 4F) VOCs, the hyphal membrane and cell walls appeared to be thinner and degraded, cytoplasmic content was completely coagulated and no organelles could be identified. These effects were also observed in *M. fructicola* hyphae treated with VOCs produced by the *P. vulpis* Z8 strain (Figure 4H).

## Discussion

Bacteria emit VOCs that can inhibit the growth of specific microbial populations (Larkin and Stokes, 1967; Chaves-Lopez et al., 2015). These VOCs, which are easily degraded and act over long distances (Gao et al., 2017), exhibit major advantages over conventional fungicides. In this study, we examined the diversity of VOCs produced by extremophilic bacteria and their antagonistic activity against certain phytopathogenic fungi. Their ability to secrete a wide range of antifungal VOCs could make these bacteria useful in the search for potential biological agents to control postharvest diseases.

Roughly 2,000 compounds produced by almost 1,000 microorganism species are listed in the MVOC database (Lemfack et al., 2018; Caulier et al., 2019). We found acetoin, acetic acid, 2,3-butanediol, isopentanol, dimethylsulfide and isopentyl isobutanoate to be the principal volatile compounds produced by the studied strains.

GC/MS analysis showed that high levels of acetoin and 2,3-butanediol are produced by *Bacillus* strains. Acetoin, which stimulates induced systemic resistance against *Pseudomonas syringae* DC3000 (Sierra, 1957) and plant growth (Kai and Piechulla, 2009) and is produced by other *Bacillus* strains (Asari et al., 2016; Caulier et al., 2019), is synthesised in all media, with particularly high levels detected in MOLP medium, while 2,3-butanediol was exclusively found in MOLP cultures. In addition, the volatile compound 2,3-butanediol is not only known to promote plant growth (Wu et al., 2019) but also to induce modifications in the expression of genes linked to *Ralstonia solanacearum* and *Pectobacterium carotovorum* pathogenicity (Marquez-Villavicencio Mdel et al., 2011; Tahir et al., 2017).

The antifungal activity of VOCs has been reported to be associated with functional groups (Sasser, 1990; Chaves-Lopez et al., 2015). In addition, solute hydrophobicity affects membrane integrity, which is undermined by the application of lipophilic compounds. This may affect DMDS, the principal volatile compound produced by Z8, and could be responsible for the strong antifungal activity of this bacterium against *Monilia* sp. and *S. sclerotium*. The antifungal activity of this volatile compound has been characterised in *Bacillus* strains (Coosemans, 2005; Caulier et al., 2019) and in *Pseudomonas donghuensis* against *Rhizoctonia solani* (Ossowicki et al., 2017; Guevara-Avendano et al., 2019). DMDS, a sulphur-containing compound produced by *Pseudomonas* sp., also inhibits *Phytophthora infestans* growth and development (De Vrieze et al., 2015; Guevara-Avendano et al., 2019).

Acetic acid is the principal volatile compound produced by strains *Peribacillus* sp. N3 and *S. equorum* EN21 when cultured in MOLP and SG media. Acetic acid has been found to be an effective antifungal compound in several fruits (Baird-Parker, 1963; Fuqua et al., 1994), in low concentrations, reduce *B. cinerea* and *P. expansum* germination to zero (Baird-Parker, 1963) and, at a concentration of 50mm significantly inhibit the growth of the phytopathogenic fungus *C. gloeosporioides* (Kang et al., 2003). The inhibition by this organic acid is closely related to reduced respiration levels rather than to structural cellular damage. Momma et al. (2006) have described how the treatments of soil with acetic acid suppress the survival of *R. solanacearum*.

In addition, high levels of isopentanol were produced by strain *S. equorum* EN21 in all media, especially in TSB. The antifungal activity of alcohols has been described in previous studies; for example, the phenol 4-chloro-3-methyl has been reported to have a strong antifungal effect on *Alternaria solani* and *B. cinerea* (Gao et al., 2017), allyl alcohol inhibits *S. sclerotiorum* germination in bean plants (Lim et al., 2017), while phenylethyl alcohol inhibits the mycelial growth of *P. italicum* (Li et al., 2010).

High concentrations of isopentyl isobutanoate/isobutyrate were only detected when strain *P. vulpis* Z8 was cultured in all media. To the best of our knowledge, no information concerning the antifungal activity of this compound, which could be responsible for the intense antifungal activity of Z8 against *M. fructicola* o *S. sclerotiorum*, existed prior to our study.

A group of highly correlated VOCs was clearly identified for all the strains investigated except for *P. vulpis* Z8 and *S. equorum* EN21. Several studies have analysed volatile metabolites emitted by clinical *Staphylococcus* sp. strains (Nasir et al., 2018) but only one has identified antifungal volatile agents produced by *Staphylococcus pasteuri* against the commercial truffle species *Tuber borchii* Vittad (Barbieri et al., 2005).

The VOC 2,6-diethyl-pyrazine produced by *S. equorum* strain EN21 is involved in segregating this bacterium from other bacteria. A large proportion of microbial volatiles is pyrazines, which are produced by many strains such *Bacillus subtilis* and are known to exhibit antifungal activity (Chaves-Lopez et al., 2015; Haidar et al., 2016; Caulier et al., 2019). Some of these compounds affect the sporulation and elongation of *B. cinerea* germ tubes (Chaves-Lopez et al., 2015; Haidar et al., 2016; Caulier et al., 2019), while 2,3,5-trimethylpyrazine antifungal activity against bacterium *Fusarium* sp. has also been studied (Guevara-Avendano et al., 2019).

The antifungal activity of bacterial volatile compounds tested *in vitro* demonstrated that strains *B. atrophaeus* L193, *B. velezensis* XT1 and *P. vulpis* Z8 cultured in MOLP medium significantly inhibited the growth of *S. sclerotiorum* and *M. fructicola*. Strains *B. atrophaeus* L193 and *B. velezensis* XT1 reduced the growth of *M. laxa* and *B. cinerea* by between 30 and 55%. Species of the genus *Bacillus* have been reported to be the most common producers of bioactive VOCs (Chaves-Lopez et al., 2015). The *in vitro* activity of VOCs against the major phytopathogenic species *B. subtilis*, *Bacillus amyloliquefaciens* and *B. velezensis* has been studied previously (Chaves-Lopez et al., 2015). However, few studies have analysed the volatilome produced by *B. atrophaeus*, and, to the best of our knowledge, this is the first study of the antifungal activity of VOCs produced by the genera *Psychrobacillus* and *Peribacillus*.

Our findings show that none of the bacteria tested were effective against all phytopathogenic fungi. Similar results were obtained by Chaves-Lopez et al. (2015) in their study of *Bacillus* strains. The variations in fungal responses could reflect differences in the sites of action or in the ability of fungi to detoxify the metabolites (Chaves-Lopez et al., 2015). Strains *Bacillus* and *Psychrobacillus*, but not *B. frigotolerans*, showed antifungal activity against major phytopathogens such *Monilinia* and *Sclerotinia*.

Previous *in vitro* studies have demonstrated that VOCs produced by *B. velezensis* inhibit the growth of *B. cinerea* and *M. fructicola* by over 70% (Gao et al., 2017; Guevara-Avendano et al., 2019), which is within the 50–97% range observed in our study of *B. velezensis* XT1 and *B. atrophaeus* L193. We found lower levels of antifungal activity against *Fusarium* for all strains tested, with a 38% reduction observed following exposure to VOCs produced by *B. atrophaeus* strain L193, which is very similar to the reduction observed for other *Bacillus* strains (Yuan et al., 2012; Guevara-Avendano et al., 2019). The bacteria which generally showed the most effective antifungal activity, *B. velezensis* XT1 and *B. atrophaeus* L193, was found to be producers of metabolites with antifungal activity, such as lipopeptides in previous studies (Rodriguez et al., 2018; Toral et al., 2018). Although little is known about VOCs produced by *B. atrophaeus*, the volatilome of *B. atrophaeus* HAB-5 has been identified which has a moderate antifungal effect on the common disease of anthracnose caused by the major fungus *C. gloeosporioides* (Rajaofera et al., 2019). No information on the antifungal activity of volatile compounds produced by *B. atrophaeus* against the major phytopathogens analysed existed prior to our study.

The bacterial genus *Pseudomonas* is also capable of emitting VOCs that inhibit the mycelial growth of *S. sclerotiorum* (Fernando et al., 2005; Guevara-Avendano et al., 2019), although the inhibition rate recorded for *P. segetis* P6 in this study was very low as compared to the other strains tested. Species of the genus *Staphylococcus* are mostly known to cause opportunistic human diseases, some of which are frequently found in rhizospheric soil. Although antifungal activity of the bacterium *S. equorum* against *B. cinerea* has been observed (Sadfi-Zouaoui et al., 2008; Reverchon et al., 2019) to our knowledge, the VOCs produced by this bacterium have not previously been described. In our study, the antifungal activity of *S. equorum* strain EN21 against *F. solani* was found to be very low, with a reduction in fungal growth of only 2%. Reverchon et al. (2019) have shown that VOCs emitted by some *Staphylococcus* species, which are capable of inhibiting the growth of *F. solani* by over 20%, can, however, increase that of *F. oxysporum*.

Overall, our results suggest that the antifungal activity of VOCs produced by the extremophilic bacteria studied is dependent on a combination of a limited number of molecules, while the composition of VOC profiles not only depends on species but also on the growing medium (Larkin and Stokes, 1967; Chaves-Lopez et al., 2015). The differences in proteins and sugar content in all media reflect differences in the volatilomes produced, whose composition and functional properties are known to be influenced by the growth medium (Asari et al., 2016; Lazazzara et al., 2017). The profiles of VOCs obtained in the present study for different *Bacillus* strains confirmed the genetic impact on their composition.

Previous studies have shown that bacterial VOCs can affect the hyphal growth, sporulation and spore germination of fungi (Wenke et al., 2010; Asari et al., 2016). Zhou et al. (2019) have suggested that VOCs produced by *B. subtilis* permeabilize fungal spores and inhibit the germination of *M. fructicola*. Other studies have highlighted how the fungal hyphae of *A. solani* are deformed when treated with VOCs produced by *B. subtilis* strains (Zhang et al., 2020b).

The effects of VOCs produced by the strains tested on the morphology of phytopathogens were evaluated using microscopic analysis. TEM micrographs of fungi treated with bacterial VOCs clearly show the impact of antifungal activity, which causes a marked deterioration in cellular components and cell death and inhibits fungal growth and development. Some studies have previously described a similar destruction of fungal structures and cell death induced by VOCs from *Pseudomonas* spp. strains USB2104 and USB2105, as well as *Bacillus* sp. USB2103, against *S. sclerotiorum* (Giorgio et al., 2015), from *B. subtilis CF*-3 against *M. fructicola* (Zhou et al., 2019) and by synthetic COVs against *B. cinerea* (Liu et al., 2016). By contrast, no antifungal activity mediated by VOCs from species of the genus *Psychrobacillus* was analysed prior to our study.

We investigated the production of several volatile metabolites with different types of biological activity. Previous studies have shown relationships between phytotoxicity and VOCs (Arimura et al., 2010). In some cases, terpenic VOCs inhibited or reduced the germination of seeds of cereals (He et al., 2014). However, exposure of *Arabidopsis thaliana* of alcoholic VOCs showed non-effect on germination (Lee et al., 2014). These studies highlighted the influence of the origin, dose and application form in the antimicrobial activity of VOCs. *In vivo* analyses described the potential of VOCs emitted by *Bacillus* sp. to control postharvest diseases (Zheng et al., 2019). The presence of residual antibiotics produced by these bacteria in fruits constitutes an important health risk because the increased microbial resistance detected in last years (Rashmi et al., 2017). However, VOCs are naturally occurring (emissions by microorganisms) at very low concentrations and do not leave toxic residues on fruit surfaces (Mercier and Smilanick, 2005; Qin et al., 2017). The biological fumigation with VOCs is an interesting strategy to use against a wide range of storage pathogens and fungal decay.

Our search for extremophilic bacteria capable of withstanding stress conditions identified several good candidate strains for postharvest use as VOCs producers. The antifungal activity of many of the VOCs identified in this work has not been previously studied, and further research is required in order to better understand the mechanistic role of VOCs produced by extreme bacteria in antifungal activity to control postharvest diseases.

## Data Availability Statement

The raw data supporting the conclusions of this article will be made available by the authors, without undue reservation.

## Author Contributions

LT, MR, FM-C, IL, and IS conceived and supervised the study and designed the experiments. LT, MR, AS, AM, and AC-D performed the experiments and analysed the data. LT, MR, FM-C, and IS prepared the figures, drafted the manuscript, and wrote the final version of the manuscript. All authors contributed to the article and approved the submitted version.

## Funding

This research was funded by grants from the Spanish Ministry of the Economy and Competitiveness (PID2019-106704RB-100/ AEI/10.13039/501100011033), the European Project for Industrial Doctorates ‘H2020’ (UGR-Ref. 4726), and B-AGR-222-UGR20 funded by Consejería de Universidad, Investigación e Innovación de la Junta de Andalucía and, ERDF A way of making Europe.

## Conflict of Interest

The authors declare that the research was conducted in the absence of any commercial or financial relationships that could be construed as a potential conflict of interest.

## Publisher’s Note

All claims expressed in this article are solely those of the authors and do not necessarily represent those of their affiliated organizations, or those of the publisher, the editors and the reviewers. Any product that may be evaluated in this article, or claim that may be made by its manufacturer, is not guaranteed or endorsed by the publisher.
