# Corrigendum: Identification of volatile organic compounds in extremophilic bacteria and their effective use in biocontrol of postharvest fungal phytopathogens

**DOI:** 10.3389/fmicb.2023.1267324

**Published:** 2023-08-10

**Authors:** Laura Toral, Miguel Rodríguez, Fernando Martínez-Checa, Alfredo Montaño, Amparo Cortés-Delgado, Agnieszka Smolinska, Inmaculada Llamas, Inmaculada Sampedro

**Affiliations:** ^1^Xtrem Biotech S.L., European Business Innovation Center, Avenida de la Innovación, Granada, Spain; ^2^Department of Microbiology, Faculty of Pharmacy, Campus de Cartuja s/n, Granada, Spain; ^3^Biomedical Research Center (CIBM), Avenida del Conocimiento s/n, Granada, Spain; ^4^Department of Food Biotechnology, Instituto de la Grasa, Sevilla, Spain; ^5^Department of Pharmacology and Toxicology, Maastricht University, Maastricht, Netherlands

**Keywords:** volatile compounds, antifungal activity, biocontrol, fungal phytopathogens, postharvest diseases

In the published article, there was an error in the Funding statement. This research was funded by grants from the Spanish Ministry of the Economy and Competitiveness (PID2019-106704RB-100/ AEI/10.13039/501100011033) and the European Project for Industrial Doctorates ‘H2020' (UGR-Ref. 4726).

The correct Funding statement appears below.

