# Supplementary material for: Identification of Volatile Organic Compounds in Extremophilic Bacteria and Their Effective Use in Biocontrol of Postharvest Fungal Phytopathogens
Source: Front Microbiol. 2021 Nov 12;12:773092. doi: 10.3389/fmicb.2021.773092 (PMC8633403; doi:10.3389/fmicb.2021.773092)
Supplement: Supplementary file 1 [file Data_Sheet_1.pdf]

Supplementary Material

Table S1. Full spectrum of volatile compounds identified by GC/MS after HS-SPME. Reference (Ref) for each compound were assigned arbitrary for further analysis. Quantification of volatile compounds were carried out by comparation of peak areas to that of internal standard (5-nonanol) and the mean of two replicates is shown in this table with SD in parenthesis. Retention time (RT) of each compound is shown and its precision is based on the experimental procedure. Identification scale of each compound (Id) is based on: 1, identified (mass spectrum and RT were in accordance with standards); 2, tentatively identified (mass spectrum matched in the standard NIST 2017 library and RT matched with literature); 3, tentatively identified (mass spectrum agreed with the standard NIST 2017). -, not detected.

Ref	Volatile compound	CAS number	RT (min)	Id	Concentration of volatile compounds (μg L^{-1})																				
					Uninoculated media			*S. equroum* EN21			*B. atrophaeus* L193			*Peribacillus* sp. N3			*P. segetis* P6			*B. velezensis* XT1			*P. vulpis* Z8		
					MOLP	SG	TSA	MOLP	SG	TSA	MOLP	SG	TSA	MOLP	SG	TSA	MOLP	SG	TSA	MOLP	SG	TSA	MOLP	SG	TSA
1	Acetone	67-64-1	2.184	1	4.69 (0.7)	4.13 (0.0)	3.19 (0.3)	28.91 (8.6)	9.33 (0.3)	22.19 (8.9)	7.81 (1.7)	4.33 (1.5)	3.33 (0.5)	10.2 (0.2)	9.00 (0.2)	1.78 (0.5)	3.1 (1.2)	3.32 (0.2)	1.56 (0.1)	6.19 (0.4)	3.9 (0.2)	3.24 (0.3)	3.73 (0.4)	4.46 (0.1)	3.57 (0.1)
2	Butanal	123-72-8	2.649	2	2.83 (0.5)	-	-	-	-	-	-	-	-	-	-	-	-	-	-	-	-	-	-	-	-
3	Ethyl Acetate	141-78-6	2.738	1	22.99 (2.8)	30.07 (0.7)	24.93 (1.8)	26.6 (5.3)	29.5 (2.3)	31.68 (13.1)	12.5 (1.0)	2.82 (0.7)	14.16 (0.3)	19.54 (0.5)	26.79 (1.6)	17.49 (1.1)	27.45 (5.4)	17.37 (1.3)	18.76 (0.8)	23.02 (2.3)	16.3 (1.1)	14.29 (0.9)	15.33 (0.8)	24.33 (0.6)	18.3 (1.0)
4	Methyl Acetate	79-20-9	2.277	1	-	0.62 (0.0)	-	-	-	-	-	-	-	-	-	-	-	-	-	-	-	-	-	-	-
5	2-Butanone	78-93-3	2.877	2	4.67 (0.6)	1.46 (0.1)	2.71 (0.5)	4.19 (0.7)	1.26 (0.3)	3.6 (1.4)	5.43 (0.2)	3.04 (0.9)	4.12 (0.0)	5.1 (0.3)	2.02 (0.2)	3.38 (0.7)	8.72 (1.9)	2.63 (0.1)	4.43 (0.2)	5.12 (0.4)	1.26 (0.0)	2.15 (0.2)	8.02 (0.1)	-	3.36 (0.2)
6	2-Methylbutanal	96-17-3	3.024	1	-	0.56 (0.0)	4.22 (0.4)	2.17 (0.3)	2.24 (0.3)	-	-	-	-	1.34 (0.5)	2.55 (0.1)	-	-	-	-	-	-	-	3.44 (1.4)	-	3.56 (0.1)
7	3-Methylbutanal	590-86-3	3.078	2	28.69 (8.0)	2.45 (0.0)	20.81 (2.0)	6.84 (0.8)	10.74 (0.8)	4.96 (2.4)	-	-	-	5.04 (1.1)	3.44 (0.3)	-	-	-	-	1.81 (0.2)	-	-	2.48 (0.6)	-	5.39 (0.2)
8	2,3-Butanedione	431-03-8	4.141	1	1.51 (0.2)	0.51 (0.0)	1.39 (0.1)	-	-	-	10.59 (1.8)	6.01 (1.5)	3.49 (1.4)	20.05 (4.7)	3.92 (0.2)	-	-	-	-	9.26 (0.1)	8.47 (0.1)	5.65 (0.2)	-	-	-
9	2-Methyl-2-butenal	1115-11-3	7.461	1	1.02 (0.0)	-	-	-	-	-	-	-	-	-	-	-	-	-	-	-	-	-	-	-	-
10	Isopentanol	123-51-3	12.490	1	-	-	-	34.32 (2.1)	73.41 (3.5)	110.63 (27.7)	1.79 (0.1)	-	-	7.68 (0.2)	1.06 (0.3)	0.77 (0.3)	2.41 (0.5)	-	3.07 (0.1)	-	-	-	6.11 (1.1)	-	-
11	Isopentyl acetate	123-92-2	8.497	1	-	-	-	4.07 (1.2)	1.60 (0.2)	3.05 (0.6)	-	-	-	0.97 (0.0)	-	-	-	-	-	-	-	-	2.11 (0.2)	-	-
12	1-Butanol	71-36-3	9.911	1	4.26 (0.2)	1.15 (0.0)	8.01 (0.3)	27.24 (2.4)	1.97 (0.2)	16.05 (4.2)	46.91 (8.0)	4.5 (0.9)	16.92 (0.1)	6.12 (0.1)	-	1.00 (0.0)	-	-	-	15.09 (0.8)	-	-	4.83 (0.6)	-	6.03 (0.3)
13	Ethanol	64-17-5	3.296	1	-	0.67 (0.0)	-	-	0.95 (0.0)	-	-	0.98 (0.2)	0.87 (0.0)	-	-	-	-	-	-	-	-	-	1.27 (0.2)	-	-
14	D-Limonene	5989-27-5	11.412	1	1.44 (0.2)	0.69 (0.1)	2.34 (1.9)	-	5.23 (0.6)	6.81 (1.9)	-	1.88 (1.3)	2.23 (1.5)	1.06 (0.1)	3.25 (2.0)	1.65 (1.9)	-	-	8.67 (1.1)	-	1.31 (0.1)	3.07 (0.7)	-	-	-
15	Pyrazine	290-37-9	12.308	2	1.61 (0.1)	-	4.59 (0.3)	2.87 (0.1)	-	5.63 (1.0)	2.78 (0.2)	-	5.9 (0.0)	1.69 (0.1)	-	3.96 (0.1)	2.88 (0.4)	-	5.12 (0.1)	2.73 (0.2)	-	5.36 (0.1)	1.32 (0.2)	-	4.87 (0.2)
16	Styrene	100-42-5	14.215	1	-	0.56 (0.1)	0.6 (0.0)	-	-	-	-	-	-	0.74 (0.3)	1.98 (0.7)	-	-	-	-	-	-	-	-	-	-
17	Pyrazine. methyl-	109-08-0	14.670	2	1.78 (0.1)	-	4.05 (0.2)	3.04 (0.1)	-	5.05 (0.7)	3.11 (0.1)	-	5.15 (0.2)	1.7 (0.1)	-	3.48 (0.1)	2.83 (0.2)	-	4.57 (0.0)	2.92 (0.2)	-	4.7 (0.0)	1.8 (0.2)	-	4.41 (0.3)
18	2-Propanone. 1-hydroxy-	116-09-6	16.206	2	0.88 (0.1)	-	1.24 (0.3)	-	-	-	-	-	-	1.5 (0.0)	-	1.21 (0.3)	1.03 (0.0)	-	1.06 (0.1)	-	-	-	-	-	-
19	Pyrazine. 2.5-dimethyl-	123-32-0	17.162	2	13.69 (0.8)	-	49.48 (3.4)	32.01 (0.3)	-	61.01 (5.2)	30.77 (1.4)	-	66.3 (0.1)	9.92 (0.2)	-	36.62 (0.6)	32.92 (5.1)	0.58 (0.0)	54.84 (0.1)	25.57 (0.3)	-	57.26 (0.1)	15.05 (0.2)	-	56.31 (0.8)
20	Pyrazine. 2-ethyl-5-methyl-	13360-64-0	20.167	2	1.26 (0.2)	-	1.75 (0.1)	2.23 (0.1)	-	2.17 (0.0)	1.95 (0.0)	-	2.38 (0.1)	0.96 (0.0)	-	1.16 (0.0)	-	-	2.18 (0.0)	2.13 (0.1)	-	2.13 (0.1)	1.15 (0.1)	-	2.01 (0.2)
21	Pyrazine. trimethyl-	14667-55-1	20.748	2	1.48 (0.1)	-	1.77 (0.2)	2.72 (0.1)	-	2.29 (0.0)	2.45 (0.2)	-	2.46 (0.1)	1.24 (0.1)	-	1.49 (0.1)	2.37 (0.3)	-	2.11 (0.0)	2.45 (0.0)	-	2.22 (0.0)	2.22 (0.2)	-	2.92 (0.3)
22	Pyrazine. 3-ethyl-2,5-dimethyl-	13360-65-1	22.467	2	1.05 (0.0)	-	1.38 (0.1)	-	-	1.72 (0.1)	1.47 (0.1)	-	1.56 (0.0)	0.84 (0.0)	-	1.04 (0.0)	1.42 (0.1)	-	1.58 (0.0)	1.54 (0.1)	-	1.53 (0.0)	3.23 (0.2)	-	4.03 (0.2)
23	2-Ethyl-1-hexanol	104-76-7	24.707	1	-	-	-	-	-	-	-	0.95 (0.3)	0.85 (0.0)	-	-	1.2 (0.0)	-	0.73 (0.1)	6.93 (0.2)	-	-	-	-	-	-
24	Acetic acid	64-19-7	22.523	1	3.75 (3.0)	-	-	180.23 (17.1)	25.58 (1.9)	4.13 (2.0)	36.85 (7.1)	10.02 (6.5)	3.56 (1.1)	32.85 (4.5)	30.8 (0.4)	1.26 (0.2)	-	-	-	21.9 (0.4)	6.07 (4.1)	2.12 (0.0)	-	-	-
25	Benzaldehyde	100-52-7	25.552	1	39.82 (5.7)	16.64 (0.6)	52.89 (3.3)	4.61 (0.4)	5.1 (0.1)	-	1.11 (0.1)	0.45 (0.2)	0.58 (0.0)	5.31 (0.4)	11.55 (1.0)	0.37 (0.1)	2.03 (1.2)	-	-	7.04 (1.2)	3.0 (0.9)	-	1.72 (0.2)	2.71 (0.4)	-
26	Ethanol. 2-(2-ethoxyethoxy)-	111-90-0	29.655	2	3.02 (0.5)	2.83 (0.2)	2.91 (0.1)	2.43 (0.3)	1.55 (0.2)	2.31 (0.8)	3.28 (1.2)	1.5 (0.1)	2.33 (0.0)	2.37 (0.1)	3.58 (1.5)	1.8 (0.1)	2.63 (0.3)	1.38 (0.2)	1.43 (0.1)	3.55 (0.4)	1.68 (0.0)	1.88 (0.2)	2.66 (0.5)	2.8 (0.6)	1.9 (0.3)
27	Butanoic acid	107-92-6	30.077	1	1.09 (0.8)	-	-	18.50 (1.3)	-	-	4.03 (1.4)	-	-	4.92 (0.4)	-	-	-	-	-	3.95 (0.5)	-	-	-	-	-
28	Acetophenone	98-86-2	30.536	2	1.33 (0.0)	-	-	1.57 (0.1)	-	-	-	-	-	1.04 (0.0)	-	-	-	-	-	1.62 (0.1)	-	-	-	-	-
29	Propanoic acid	79-09-4	26.597	2	-	-	-	3.54 (0.3)	-	-	-	-	-	1.22 (0.2)	0.9 (0.2)	-	-	-	-	-	-	-	-	-	-
30	Isobutyric acid	79-31-2	27.776	1	-	-	-	5.66 (0.6)	1.92 (0.2)	-	-	1.61 (0.7)	-	5.4 (0.2)	4.76 (0.6)	-	-	-	-	-	1.03 (0.4)	-	3.45 (0.1)	1.95 (0.1)	-
31	Propanoic acid, 2-methyl-, 3-hydroxy-2,2,4-trimethylpentyl ester	77-68-9	38.727	3	3.20 (1.7)	3.22 (0.8)	1.05 (0.1)	-	-	-	-	-	-	3.16 (0.9)	-	-	-	-	-	-	-	-	2.95 (0.6)	-	-
32	2,2,4-Trimethyl-1,3-pentanediol diisobutyrate	6846-50-0	39.014	3	7.24 (0.2)	6.48 (0.5)	4.8 (4.8)	-	-	-	-	-	-	4.48 (1.2)	-	-	1.22 (0.3)	-	-	-	1.17 (0.3)	-	-	-	-
33	Butyl acetate	123-86-4	6.763	2	-	-	-	2.43 (0.4)	-	0.78 (0.2)	-	-	-	0.99 (0.1)	-	-	-	-	-	-	-	-	-	-	-
34	1,3-Di-tert-butylbenzene	1014-60-4	21.849	2	-	-	-	-	-	2.26 (0.1)	5.49 (0.4)	1.47 (0.1)	5.46 (0.4)	-	1.14 (0.2)	1.1 (0.0)	1.28 (0.0)	-	0.47 (0.1)	4.98 (0.9)	3.09 (0.0)	1.87 (0.4)	2.17 (0.4)	-	2.18 (0.1)
35	Pyrazine. 2,6-diethyl-	13067-27-1	22.605	2	-	-	-	1.64 (0.1)	-	-	-	-	-	-	-	-	-	-	-	-	-	-	-	-	-
36	2-Acetylthiazole	24295-03-2	30.447	2	-	-	-	2.20 (0.1)	-	-	-	-	-	-	-	-	-	-	-	-	-	-	-	-	-
37	3-Methylbutanoic acid	503-74-2	31.666	1	-	-	-	14.95 (1.5)	21.29 (2.7)	5.73 (0.9)	8.44 (1.6)	8.79 (3.4)	3.71 (1.4)	11.74 (0.5)	16.11 (2.3)	1.74 (0.2)	-	-	-	3.44 (0.4)	6.80 (0.5)	3.4 (0.3)	9.08 (1.6)	1.13 (0.1)	2.25 (0.1)
38	Hexanoic acid	142-62-1	38.020	1	-	-	-	3.25 (0.2)	-	-	-	-	-	0.74 (0.3)	-	-	-	-	-	-	-	-	-	-	-
39	Octanoic acid	124-07-2	45.104	1	-	-	-	3.05 (0.1)	-	-	-	-	-	0.86 (0.0)	-	-	-	-	-	-	-	-	-	-	-
40	Nonalal	124-19-6	20.509	1	-	-	-	-	2.76 (1.5)	-	-	-	-	-	-	-	-	-	-	-	-	-	-	-	-
41	Phenylethyl Alcohol	60-12-8	39.958	1	-	-	-	-	1.61 (0.2)	4.17 (0.2)	0.65 (0.0)	-	-	1.37 (0.0)	-	-	-	-	-	-	-	-	-	-	-
42	2-Pentanone	107-87-9	4.055	2	-	-	-	-	-	1.32 (0.2)	1.28 (0.0)	-	-	-	-	-	0.79 (0.3)	-	1.28 (0.1)	3.27 (0.4)	-	2.44 (0.1)	-	-	-
43	2-Heptanone	110-43-0	11.066	2	-	-	-	-	-	-	16.27 (0.7)	3.09 (0.2)	5.28 (0.3)	-	-	-	-	-	-	6.57 (0.7)	1.16 (0.0)	2.98 (0.3)	-	-	-

44	5-Methyl-2-heptanone	18217-12-4	14.289	2	-	-	-	-	-	-	1.98 (0.0)	1.78 (0.2)	1.45 (0.1)	-	-	-	-	-	-	-	-	-	-	-	-
45	Acetoin	513-86-0	15.655	1	-	-	-	-	-	-	174.03 (10.4)	60.53 (15.6)	44.47 (0.3)	2.59 (0.0)	-	-	0.73 (0.1)	-	-	110.99 (8.5)	64.02 (9.6)	60.03 (0.5)	-	-	-
46	2,3-Butanediol	24347-58-8	26.896	1	-	-	-	-	-	-	268.83 (23.0)	-	-	-	-	-	-	-	-	261.74 (49.3)	-	-	-	-	-
47	o-Guaiacol	90-05-1	38.244	1	-	-	-	-	-	-	1.47 (0.1)	-	-	-	-	-	-	-	-	-	-	-	-	-	-
48	Phenol	108-95-2	43.221	1	-	-	-	-	-	-	3.74 (0.3)	-	-	-	-	-	-	-	-	-	-	-	-	-	-
49	3-Methyl-2-pentanone	565-61-7	4.949	2	-	-	-	-	-	-	-	1.43 (0.4)	1.44 (0.0)	-	-	-	-	-	-	-	-	-	-	-	-
50	6-Methyl-2-heptanone	928-68-7	13.455	2	-	-	-	-	-	-	-	1.02 (0.1)	0.59 (0.0)	-	-	-	-	-	-	-	-	-	-	-	-
51	3-Hydroxy-3-methyl-2-butanone	115-22-0	13.877	2	-	-	-	-	-	-	-	1.05 (0.1)	1.45 (0.1)	-	-	-	-	-	-	-	1.12 (0.0)	1.24 (0.2)	-	-	-
52	2-Ethylbutanoic acid	88-09-5	19.762	1	-	-	-	-	-	-	-	7.71 (1.3)	-	-	-	-	-	-	-	-	4.54 (0.3)	2.55 (0.2)	-	-	-
53	1-Octanol	111-87-5	27.443	1	-	-	-	-	-	-	-	0.64 (0.1)	-	-	-	-	-	-	-	-	-	-	-	-	-
54	Trichloromethane	67-66-3	5.139	2	-	-	-	-	-	-	-	-	-	1.25 (0.1)	1.75 (0.0)	0	-	-	-	-	-	-	-	-	-
55	Octyl octanoate	2306-88-9	43.707	2	-	-	-	-	-	-	-	-	-	2.37 (0.1)	-	-	-	-	-	-	-	-	-	-	-
56	n-Decanoic acid	334-48-5	51.608	1	-	-	-	-	-	-	-	-	-	0.52 (0.1)	-	-	-	-	-	-	-	-	-	-	-
57	Dimethyl disulfide	624-92-0	6.635	2	-	-	-	-	-	-	-	-	-	-	1.34 (0.0)	5.56 (1.8)	3.61 (0.3)	46.08 (2.9)	1.7 (0.0)	-	-	-	88.41 (2.7)	139.67 (1.5)	140.12 (4.3)
58	Phenethyl 2-methylbutyrate	24817-51-4	42.050	2	-	-	-	-	-	-	-	-	-	-	1.39 (0.3)	-	-	-	-	-	-	-	47.95 (3.2)	1.31 (0.0)	13.95 (1.2)
59	Benzoic acid	65-85-0	55.707	1	-	-	-	-	-	-	-	-	-	-	2.26 (0.2)	-	-	-	-	-	-	-	-	-	-
60	Methanethiol	74-93-1	1.716	2	-	-	-	-	-	-	-	-	-	-	-	1.64 (0.7)	-	-	-	-	-	-	12.54 (3.3)	6.73 (0.9)	9.79 (0.6)
61	2-Butanol	78-92-2	5.277	1	-	-	-	-	-	-	-	-	-	-	-	0.56 (0.3)	-	-	-	-	-	-	-	-	-
62	Methyl thiolacetate	1534-08-3	5.935	2	-	-	-	-	-	-	-	-	-	-	-	2.24 (0.5)	-	-	-	-	-	-	10.4 (0.7)	14.73 (0.5)	6.62 (1.3)
63	4-Methyl-2-pentanone	108-10-1	4.737	2	-	-	-	-	-	-	-	-	-	-	-	-	7.28 (1.6)	4.32 (0.1)	29.84 (0.4)	-	-	-	-	-	-
64	Dimethyl sulfide	75-18-3	6.612	1	-	-	-	-	-	-	-	-	-	-	-	-	2.32 (0.8)	8.94 (0.2)	3.01 (0.1)	-	-	-	-	-	-
65	2-Nonanone	821-55-6	20.253	2	-	-	-	-	-	-	-	-	-	-	-	-	26.18 (0.7)	-	1.60 (0.3)	1.49 (0.1)	-	1.89 (0.0)	-	-	-
66	2-Nonanol	623-93-8	24.348	2	-	-	-	-	-	-	-	-	-	-	-	-	1.54 (0.3)	-	-	1.45 (0.0)	-	-	-	-	-
67	Dimethyl trisulfide	3658-80-8	19.301	2	-	-	-	-	-	-	-	-	-	-	-	-	-	0.57 (0.1)	-	-	-	-	2.36 (0.4)	3.81 (0.8)	2.03 (0.2)
68	4-Methyl-2-pentanol	108-11-2	10.727	2	-	-	-	-	-	-	-	-	-	-	-	-	-	-	0.51 (0.0)	-	-	-	-	-	-
69	2-Undecanone	112-12-9	28.964	2	-	-	-	-	-	-	-	-	-	-	-	-	-	-	-	1.36 (0.1)	-	1.57 (0.2)	-	-	-
70	2-Tridecanone	593-08-8	36.807	2	-	-	-	-	-	-	-	-	-	-	-	-	-	-	-	2.63 (0.1)	-	-	-	-	-
71	2-Dodecanone	6175-49-1	31.135	2	-	-	-	-	-	-	-	-	-	-	-	-	-	-	-	-	-	1.17 (0.3)	-	-	-
72	Ethyl isobutanoate	97-62-1	3.864	2	-	-	-	-	-	-	-	-	-	-	-	-	-	-	-	-	-	-	1.71 (0.3)	4.86 (0.1)	-
73	Ethyl 2-methylbutanoate	7452-79-1	6.093	2	-	-	-	-	-	-	-	-	-	-	-	-	-	-	-	-	-	-	1.83 (0.3)	0.75 (0.1)	-
74	Isobutyl isobutanoate	97-85-8	7.330	2	-	-	-	-	-	-	-	-	-	-	-	-	-	-	-	-	-	-	5.83 (0.3)	7.46 (0.1)	-
75	S-Methyl thiopropanoate	5925-75-7	8.332	2	-	-	-	-	-	-	-	-	-	-	-	-	-	-	-	-	-	-	5.38 (0.4)	36.72 (1.2)	13.00 (0.7)
76	S-Methyl thio-2-methylpropanoate	42075-42-3	8.860	2	-	-	-	-	-	-	-	-	-	-	-	-	-	-	-	-	-	-	13.49 (0.7)	31.48 (0.9)	7.18 (0.2)
77	Butyl propanoate	590-01-2	9.334	2	-	-	-	-	-	-	-	-	-	-	-	-	-	-	-	-	-	-	2.78 (0.2)	1.28 (0.2)	-
78	Butyl isobutanoate	97-87-0	9.566	2	-	-	-	-	-	-	-	-	-	-	-	-	-	-	-	-	-	-	29.63 (0.9)	16.91 (0.3)	18.73 (0.8)
79	Isobutyl 2-methylbutanoate	2445-67-2	10.708	2	-	-	-	-	-	-	-	-	-	-	-	-	-	-	-	-	-	-	5.27 (0.2)	-	-
80	Isopentyl isobutanoate	2050-01-3	11.615	2	-	-	-	-	-	-	-	-	-	-	-	-	-	-	-	-	-	-	82.5 (1.3)	27.41 (1.9)	51.96 (1.1)
81	S-Methyl thio-3-methylbutanoate	23747-45-7	12.818	2	-	-	-	-	-	-	-	-	-	-	-	-	-	-	-	-	-	-	23.71 (0.9)	3.31 (0.4)	8.08 (0.4)
82	Butyl 2-methylbutanoate	15706-73-7	13.277	2	-	-	-	-	-	-	-	-	-	-	-	-	-	-	-	-	-	-	16.69 (0.3)	-	6.78 (0.4)
83	Isopentyl 2-methylbutanoate	27625-35-0	15.340	2	-	-	-	-	-	-	-	-	-	-	-	-	-	-	-	-	-	-	42.62 (3.9)	-	18.84 (0.8)
84	3-(Methylthio)propyl 2-methylbutanoate	1000367-09-9	35.745	3	-	-	-	-	-	-	-	-	-	-	-	-	-	-	-	-	-	-	3.56 (0.1)	-	-
85	2-Phenylethyl isobutanoate	103-48-0	39.037	2	-	-	-	-	-	-	-	-	-	-	-	-	-	-	-	-	-	-	39.6 (5.7)	8.36 (1.5)	22.43 (0.0)
86	Isopentyl propanoate	105-68-0	11.426	2	-	-	-	-	-	-	-	-	-	-	-	-	-	-	-	-	-	-	-	1.91 (0.8)	-
87	S-Methyl pentanethioate	42075-43-4	12.360	3	-	-	-	-	-	-	-	-	-	-	-	-	-	-	-	-	-	-	-	-	7.60 (0.2)

Fig S1.

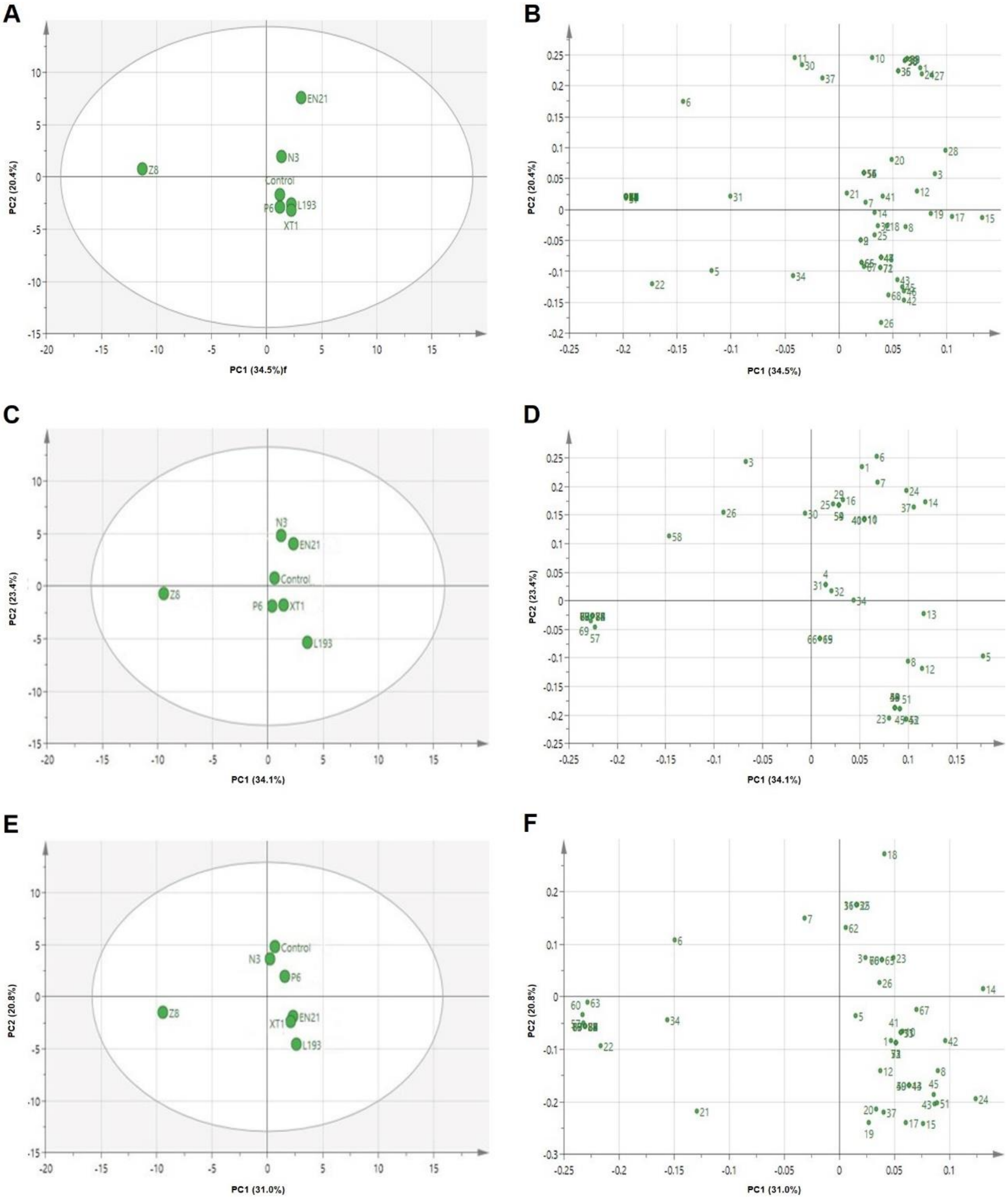
A
B
C
D
E
F
PC1 (34.5%)f
PC2 (20.4%)
PC1 (34.5%)
PC1 (34.1%)
PC2 (23.4%)
PC1 (31.0%)
PC2 (20.8%)
EN21
N3
Z8
Control
P6
L193
XT1

Fig S2.

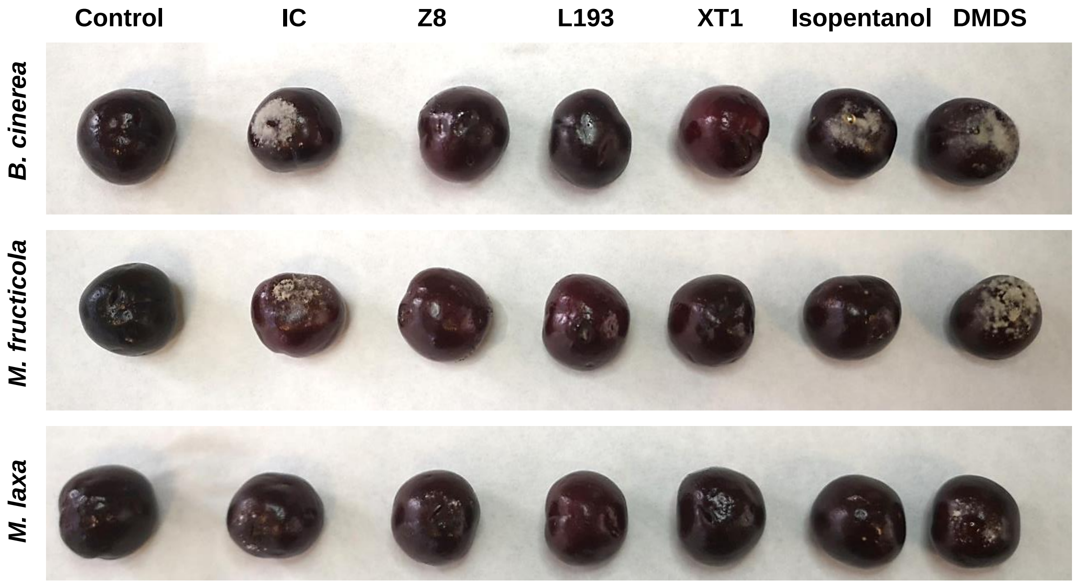
Control
IC
Z8
L193
XT1
Isopentanol
DMDS
B. cinerea
M. fructicola
M. laxa

Figure S1. Principal component analysis (PCA) of volatile compounds obtained by HS-SPME and identified by GC/MS in *B. atrophaeus* L193 strain (L193), *B. velezensis* XT1 strain (XT1), *Peribacillus sp.* N3 strain (N3), *P. segetis* strain P6 (P6), *S. equorum* subsp. *equorum* EN21 strain (EN21) and *P. vulpis* Z8 strain (Z8) grouped by culture medium. (A-B) Score and loading plots of MOLP medium. (C-D) Score and loading plots of SG medium. (E-F) Score and loading plots of TSA medium. Each volatile compound is assigned to a reference number and can be checked in table S1.

Figure S2. Effects of the volatile organic compounds produced by *P. vulpis* Z8, *B. atrophaeus* L193 and *B. velezensis* XT1 cultured in MOLP medium and the synthetic compounds isopentanol and dimethyl disulphide (DMDS) on cherry fruits artificially inoculated with *M. laxa*, *M. fructicola* and *B. cinerea*. Control: control treatment, without bacterial VOCs or volatile synthetic compounds. IC: pathogen infection control, without VOCs treatment.
